# Asprosin: A Novel Player in Metabolic Diseases

**DOI:** 10.3389/fendo.2020.00064

**Published:** 2020-02-19

**Authors:** Mingyang Yuan, Weidong Li, Yan Zhu, Boyao Yu, Jing Wu

**Affiliations:** ^1^Department of Endocrinology, Xiangya Hospital, Central South University, Changsha, China; ^2^Hunan Engineering Research Center for Obesity and Its Metabolic Complications, Changsha, China; ^3^National Clinical Research Center for Geriatric Disorders, Xiangya Hospital, Central South University, Changsha, China

**Keywords:** asprosin, metabolic diseases, diabetes, obesity, PCOS, CVD

## Abstract

Asprosin, a novel glucogenic adipokine, is encoded by two exons (exon 65 and exon 66) of the gene *Fibrillin 1* (*FBN1*) and mainly synthesized and released by white adipose tissue during fasting. Asprosin plays a complex role in the central nervous system (CNS), peripheral tissues, and organs. It is involved in appetite, glucose metabolism, insulin resistance (IR), cell apoptosis, etc. In this review, we will summarize the newly discovered roles of asprosin in metabolic diseases including diabetes, obesity, polycystic ovarian syndrome (PCOS), and cardiovascular disease (CVD), which may contribute to future clinical diagnosis and treatment.

## Introduction

Asprosin, encoded by two exons (exon 65 and exon 66) of the gene *Fibrillin 1* (*FBN1*), was first discovered as a novel glucogenic protein adipokine by Romere et al. in a study of neonatal premature aging (NPS) patients in 2016. The NPS patients, who lack asprosin attributed to a truncating mutation in *FBN1*, maintained euglycemia despite their significantly lower plasma insulin levels. Furthermore, NPS patients consumed less food, and they were extremely lean. All these abnormalities indicate the possible impacts of asprosin on carbohydrate and lipid metabolism (1). Metabolic diseases, including diabetes, obesity, PCOS, and CVD, are mainly attributed to the disruption of normal metabolic processes, posing a significant threat to human health. Recent studies have found that asprosin plays an essential and complex role in metabolism and metabolic diseases. This review will summarize the possible central and peripheral effects of asprosin and the association between asprosin and metabolic disorders including diabetes, obesity, PCOS, CVD based on recent findings. In addition, this article will also discuss asprosin as a potential therapeutic target for these diseases.

## Asprosin Synthesis and Signaling

*FBN1*, with 235 kb in length and a total of 66 exons, is located in chromosome 15q21.1 (2). These exons encode a proprotein with 2871 amino acids in length. Subsequently, the translated proprotein is cleaved at its C terminus by activated protease furin, which generates mature fibrillin-1, and 140-amino-acid long asprosin (3, 4). The white adipose tissue is a major source of asprosin; nevertheless, whether asprosin is only derived from white adipocytes is not clear, given that *FBN1* mRNA is highly expressed in several organs including the lung, the heart, etc. (1). In addition, β-cells can also excrete asprosin under hyperlipidemic conditions (5). After its synthesis, asprosin is released into the blood and shows an increased plasma concentration under fasting conditions. In addition to acting on peripheral target tissues, asprosin can also cross the blood-brain barrier and have an effect on the CNS (1, 6).

**Figure 1:**
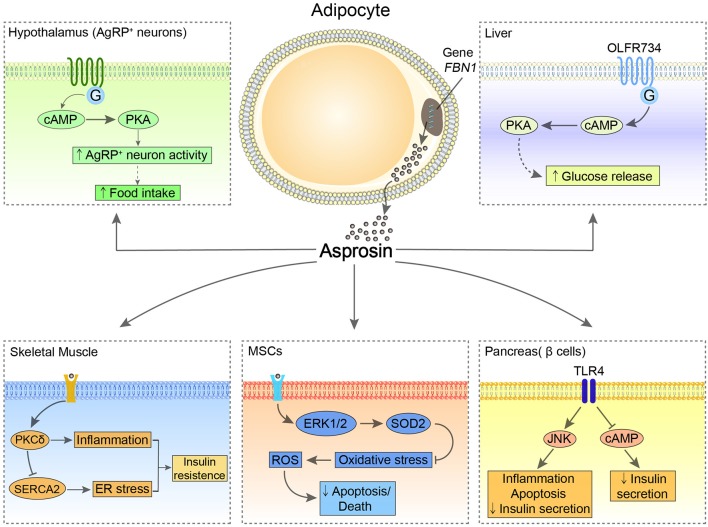
The possible central and peripheral effects of asprosin. In the hypothalamus, asprosin enhances the activity of AgRP neurons through activating G proteins-cAMP-PKA pathway, leading to increased appetite. In the liver, asprosin binds to OLFR734 to enhance the production and release of glucose via CREB pathway. Of note, the orexigenic and glucogenic roles of asprosin remain controversial. In the pancreatic β-cells, asprosin binds to TLR4 to promote inflammation and apoptosis of these cells and reduce insulin secretion through stimulating TLR4/JNK-mediated pathway and inhibiting cAMP levels. In MSCs, asprosin prevents these cells from oxidative stress-induced apoptosis and death by activating ERK1/2-SOD2 pathway. In the skeletal muscle, asprosin activates PKCδ/SERCA2-mediated ER stress/inflammation pathways to promote IR.

Asprosin has been reported to have multiple effects on CNS and peripheral tissues and organs (Figure 1). Via multiple downstream signaling pathways, asprosin has an essential impact on regulating appetite, glucose metabolism, IR, cell apoptosis, etc. (1, 5–9).

The central receptors of asprosin are mainly located in the hypothalamic arcuate nucleus, contributing to promoting appetite. As the feeding control center, the hypothalamus regulates appetite relying on two types of neuronal populations, the anorexigenic pro-opiomelanocortin (POMC) neurons and the orexigenic agouti-related peptide (AgRP) neurons (10). Asprosin increases the amplitude of AgRP neurons and changes their membrane potential, which enhances the activity of the AgRP neurons by a G proteins-cAMP-PKA axis. At the same time, this signaling inhibits the activity of POMC neurons in a GABA-dependent way, thereby stimulating food intake and regulating energy homeostasis (6).

In the periphery, the main target organs and tissues are the liver, pancreas, skeletal muscle, and heart. It has been reported that the biological effect of asprosin on the liver depends on G protein-coupled receptor (GPCR), which activates the adenylyl cyclase-PKA-cAMP responsive element binding (CREB) pathway and leads to the production and release of glucose (1). Recently, olfactory receptor 734 (OLFR734) has been determined to be one of the receptors of asprosin to mediate this effect; nevertheless, it is not the sole receptor responsible for asprosin glucogenic function (11). By binding with the unknown receptors expressed at mouse skeletal muscle cells, asprosin impairs insulin sensitivity via activating PKCδ/SERCA2-mediated endoplasmic reticulum (ER) stress/inflammation pathways. However, ROS generation, which is commonly known as a risk factor for IR, is not affected by asprosin in skeletal muscle (8). Interestingly, asprosin is able to bind to Toll-like receptor 4 (TLR4) through TLR4/JNK-mediated pathway to increase ROS production and pro-inflammatory cytokines, and thus promotes inflammation and apoptosis of β-cells (5). Conversely, asprosin may play a protective role in the heart. Asprosin significantly inhibits malondialdehyde (MDA) and reactive oxygen species (ROS) generation in glucose-induced apoptotic mouse cardiomyocytes (MCMs), and accordingly rescues these cells (9). Furthermore, by pretreatment of asprosin, myocardial mesenchymal cells (MSCs) can be protected from oxidative stress-induced apoptosis via activating ERK1/2-SOD2 pathway (7). However, the specific receptors of asprosin in these tissues and organs remain to be fully elucidated.

What should be worth noticing is that the effects of asprosin on glucogenesis and appetite remain controversial (1, 6, 8, 11, 12). A pre-clinical replication study by von Herrath et al. failed to confirm the glucogenic and orexigenic effects of asprosin reported by the group of Chopra. This study identified that poor reagent quality could be an underlying factor. As *E. coli*-derived His-asprosin was used by the Chopra's team, the difficulties to completely purify the product and the tendency for bacterial protein to aggregate could be the issues for their study (1, 12). Additionally, an *in vivo* study by Jung et al. did not confirm the orexigenic function of asprosin. The study indicated that food intake and body weight of mice were not altered by the administration of recombinant asprosin, which might be attributed to inadequate asprosin treatment period or distinct experimental conditions (8). On the other hand, the gluconeogenic and appetite-stimulated effects of asprosin were confirmed separately by two studies. First, Li et al. confirmed the gluconeogenic effect of asprosin and discovered one of the receptors responsible for it by utilizing more soluble GST-asprosin. Of note, they noted that the activity of recombinant asprosin depended heavily on its quality, and it was hard to purify His-asprosin from *E. coli*, which is consistent with the findings from Li et al. (11) and von Herrath et al. (12). Second, Chopra's team published another study and confirmed that a single dose of asprosin injected subcutaneously, either bacterially derived or mammalian-expressed, did cross the blood-brain barrier and promote food intake in mice (6). Taken together, the causes of these differences are not known and deserve to be identified. The way to produce asprosin must be considered to resolve the controversies on the metabolic role of asprosin.

How asprosin interacts with other glucogenic and appetite-related hormones in mediating hepatic glucose production and food intake has not been fully elucidated. Hormones, including glucagon, epinephrine, norepinephrine, and glucocorticoids, are known to promote glucose release from hepatocytes. Interestingly, Romere et al. noted that the treatment of asprosin exerts no impact on the levels of glucogenic hormones above. Furthermore, it is commonly known that the glucogenic effects of glucagon can also be achieved through activating G protein-cAMP-PKA axis; inhibiting the receptor of glucagon, however, has no impacts on the asprosin glucogenic influences on hepatocytes (1). This indicates asprosin plays a relatively independent role in promoting glucose release. As for appetite mediating effects, a study by the same team demonstrated the link between asprosin and ghrelin, a well-known orexigenic hormone released from the stomach. The findings revealed that a fractional overlapping subset of AgRP neurons could be activated by both asprosin and ghrelin. Furthermore, the deficiency of asprosin resulted in a reduced ability of ghrelin to activate AgRP neurons. Ghrelin receptor, on the other hand, is dispensable for the ability of asprosin to activate AgRP neurons. In addition, they reported that the mechanisms of asprosin on affecting AgRP and POMC neurons are different from an anorexigenic hormone, leptin, given that the effect of reducing food intake by neutralizing asprosin with antibodies is not affected in the absence of leptin signaling (6). Altogether, further investigations are required to uncover the crosstalk between asprosin and other hormones in the energy homeostasis system, and how expression and secretion of asprosin are affected by specific hormones and metabolites.

## Asprosin in Diabetes

There are four broad categories of diabetes: type 1 diabetes, type 2 diabetes, gestational diabetes mellitus (GDM), and other specific types of diabetes (13). Several published studies have focused on the links between the concentrations of circulating asprosin and IR as well as diabetes in mice and in humans. It has been reported that increased plasma asprosin levels can be observed in human subjects and mice with IR (1). A cross-sectional study included 143 participants who were divided into three groups: normal glucose regulation (NGR), impaired glucose regulation (IGR), and newly diagnosed type 2 diabetes mellitus (nT2DM). The study demonstrated that higher plasma asprosin was found in IGR and nT2DM groups in comparison with the NGR group, notably in IGR subjects. Asprosin was positively associated with the homeostasis model assessment for IR (HOMA-IR), while negatively related to the homeostasis model assessment for β-cell function (HOMA-β) (14). In addition, a hospital-based case-control study including 170 subjects also showed higher serum asprosin concentrations in adults with T2DM when compared with controls, and independent association between fasting glucose and serum asprosin in T2DM, which is consistent with numerous studies (14–17). Furthermore, there is an impaired or blunted response of serum asprosin levels to the fluctuation of blood glucose in type 1 diabetes mellitus (T1DM) and T2DM patients (16, 18). Recently, it has been reported that the serum asprosin levels of pregnant women with GDM and their newborns' umbilical cords are higher than controls (19). Another study found increased hepatic asprosin levels on T1DM mice as well (20). Thus, serum asprosin, which could be a biomarker, may contribute to the early diagnosis of diabetes.

The essential pathological features of T2DM are the increased fasting and postprandial blood glucose caused by the continued excess release of glucose from the liver, IR, and β-cell dysfunction (21, 22). Romere et al. noticed that a single dose of recombinant asprosin injected subcutaneously in mice led to hyperglycemia and hyperinsulinemia; neutralization of asprosin by asprosin-specific antibodies resulted in ameliorated IR and dropped plasma glucose, insulin levels as well as body weight. This suggested that the depletion of asprosin might represent a therapeutic strategy against T2DM (1). An *in vitro* study demonstrated the impact of asprosin on inflammation and changes in the function of pancreatic β-cells. This study found that palmitate augmented the secretion of asprosin in mouse insulinoma MIN6 cells, and asprosin resulted in inflammation, cellular dysfunction, apoptosis, and reduced glucose-induced insulin production in β-cells via upregulating TLR4/JNK-mediated pathway. Interestingly, these changes could be reversed through siRNA-mediated suppression of asprosin (5). The role that asprosin plays in inflammation, ER stress, and IR in skeletal muscle has been researched recently. The *in vitro* and *in vivo* results of this report demonstrated that asprosin treatment leads to skeletal muscle insulin sensitivity impairment and mice glucose and insulin tolerance deterioration through PKCδ-associated ER stress/inflammation pathways. It was indicated by the fact that treating myocytes with asprosin for 24 h and mice with recombinant mouse asprosin intraperitoneally deteriorates the insulin signaling, including decreased insulin-induced IRS-1 and Akt phosphorylation. However, the effects induced by asprosin are decreased by silencing PKCδ expression by siRNA (8). A recent study in mice with T1DM, induced by streptozotocin (STZ), revealed that aerobic exercise training reduced hepatic asprosin levels, and thus resulted in the amelioration of diabetes-related parameters. Hence, it might be helpful for the treatment of TIDM by regulating the levels of asprosin or pathways downstream of asprosin (20). Taken together, there is an apparent association between asprosin and the development of diabetes, and asprosin may provide a novel target for future management and treatment of diabetes. Of note, the causality between elevated asprosin levels and diabetes cannot be established, since whether increased asprosin is the protective feedback mechanism for diabetes or the result of metabolic disorders requires further investigations.

## Asprosin in Obesity

Latest research has found that asprosin plays a crucial and conflicting role in obesity. Numerous studies have reported increased asprosin concentrations in humans and mice with obesity. It has been reported that serum asprosin levels are pathologically elevated in obese adults, children, and mice, while reduced body weight and food intake could be observed in obese mice via using an asprosin-specific antibody (6, 23, 24). Furthermore, a positive association can be seen between circulating asprosin concentrations and waist circumference and triglyceride (TG) (14, 24). Interestingly, another study noted that human salivary glands were able to synthesize asprosin. As body mass index (BMI) of subjects increased, low-density lipoprotein cholesterol (LDL-C) and asprosin values in saliva and blood increased as well (25). Similarly, a prospective cohort study including 117 obese subjects with a BMI >35 kg/m^2^ and 57 normal participants found fasting asprosin levels were markedly higher in obese participants than in controls. In addition, the study revealed that patients with higher circulation asprosin before bariatric surgery showed greater reductions in body weight at 6 months following surgery. Specifically, this study found that the pre-surgical asprosin levels in effective-responder patients (the percentage of body weight loss within 6 months after surgery >55%) were significantly higher than non-responders (the percentage of body weight loss within 6 months after surgery <35%). The sensitivity and specificity for asprosin levels to predict effective-responders were 76% and 75%, respectively (23). Interestingly, a study by Ma et al. investigated the therapeutic effects of AM6545, a peripheral cannabinoid receptor neutral antagonist, on hypometabolic and hypothalamic obesity induced by monosodium glutamate (MSG). They revealed that AM6545 was able to decrease serum asprosin levels, which may accordingly lead to the amelioration of obesity and IR in MSG mice (26). Altogether, asprosin may be a biomarker to indicate adipose tissue mass and a target in the treatment of obesity; however, the observational studies are not able to confirm the cause-and-effect association between asprosin and obesity, and further *in vitro* and *in vivo* research needs to be done. Other studies, however, have reported contradictory effects. Jung et al. confirmed that, distinct from a previous study, whole body weight of mice did not change with recombinant asprosin administration (8). Another cross-sectional study showed that serum asprosin concentrations markedly decreased in obese 6- to 14-year-old children compared to healthy normal-weight children. Asprosin was negatively associated with BMI if adjusted for age and sex, which is not consistent with the results of the previously mentioned studies; this indicates the complex role of asprosin in obesity (27).

The mechanism by which asprosin affects obesity has not been fully elucidated. NPS patients and mice with NPS-associated mutation showed decreased plasma asprosin, appetite, and extreme leanness (1, 6). This suggested that asprosin might contribute to obesity partly through affecting appetite. The study mentioned before showed that serum asprosin was able to cross the blood-brain barrier and led to AgRP neurons being activated by a cAMP-dependent pathway to stimulate starvation, which might result in excess energy uptake and obesity (6).

## Asprosin in PCOS

PCOS is a disorder of endocrine and metabolism, which is most common in premenopausal women. PCOS is often related to IR, abdominal obesity, cardiovascular risk factors, and metabolic disorders. The main clinical manifestations are high androgen levels, ovarian dysfunction, and metabolic dysfunction (28).

Li et al. studied plasma asprosin levels in 41 women with PCOS and found that PCOS patients had markedly higher plasma asprosin concentrations than healthy subjects. There was a positive correlation between plasma asprosin level and hemoglobin A1c (HbA1c), ApoB, LDL-C, and testosterone. Besides, the study found that plasma asprosin level was an independent risk factor for PCOS through binary logistic regression analysis (17). Similarly, the positive links between asprosin and PCOS have been reported in another study (29). However, recent research reported that the metabolic characteristics of PCOS patients differed significantly from those of diabetes mellitus patients. The finding suggested that the levels of irisin were abnormal in PCOS patients, while the exact links between plasma asprosin levels and the metabolic manifestation in patients with PCOS could not be observed (30). Together, whether there is a correlation between asprosin and POCS and whether asprosin can be used as a biomarker for predicting and diagnosing PCOS remain to be further studied.

## Asprosin in CVD

Ischemic heart disease (IHD) is a disease with a high mortality rate, accounting for 12.7% of the global mortality rate (31). Thus, if the risk of the disease can be predicted, the mortality rate of the disease may be reduced significantly. The Syntax score is a grading system for evaluating the severity of angiographic vessel-specific disease (32). By comparing the levels of asprosin and the scoring system, a study showed that the changes in the levels of asprosin were positively correlated with the Syntax score. This suggested that asprosin could be a viable marker for unstable angina pectoris (UAP), and the severity of acute coronary syndrome (ACS) with UAP might be predicted by it (33).

Asprosin may play a protective role in CVD. In order to ascertain the association between serum asprosin levels and diabetic cardiomyopathy (DCM), an *in vitro* study on MCMs found that asprosin can prevent cardiomyocytes under high glucose conditions from apoptosis via reducing the production of MDA and ROS. This indicated a possible self-protection mechanism for DCM (9). Pretreatment strategies to promote stem cell functions before transplantation can improve the efficacy of treatment (34). An *in vitro* and *in vivo* study focused on the effects of asprosin on mesenchymal stromal cells (MSCs) and the effects of asprosin pretreatment on myocardial infarction (MI). The study showed that the pretreatment of asprosin on MSCs augmented homing of these cells, improved myocardial ejection function, and reduced myocardial remodeling after MI. Interestingly, asprosin did not affect the proliferation and migration of MSCs, but these cells were protected from hydrogen peroxide (H_2_O_2_)-induced injury and apoptosis through activating ERK1/2-SOD2 pathway. Therefore, asprosin can enhance the functions of MSCs and the therapeutic effects of MSCs on MI (7).

## Conclusions

**Figure 2:**
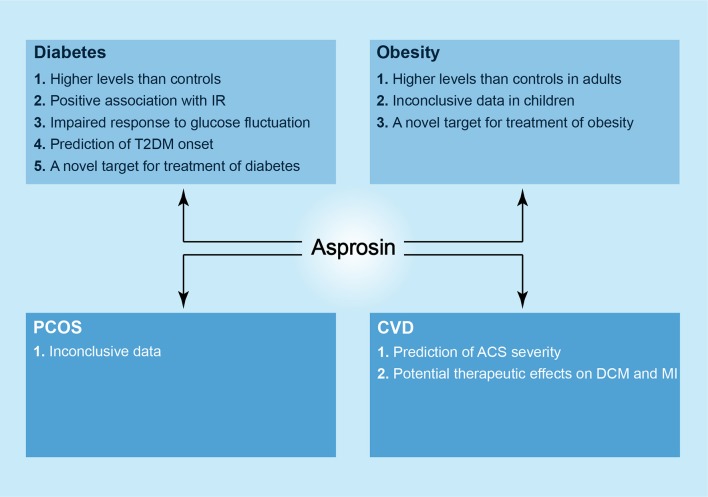
The newly discovered roles of asprosin in metabolic diseases. In diabetes, higher levels of asprosin can be observed in patients as compared to controls, and there is a positive association between serum asprosin levels and IR. Asprosin seems to show an impaired response to glucose fluctuation in both T1DM and T2DM patients. In addition, asprosin may act as a biomarker to predict T2DM onset and a novel target in the treatment of diabetes. In obesity, likewise, there are higher levels of asprosin in adult patients in comparison with controls, and asprosin may be a therapeutic target against obesity. However, the correlation between asprosin and obese children is considered as inconclusive. In PCOS, asprosin shows an inconclusive relationship to it. In CVD, asprosin may predict the severity of ACS, and it presents a possible strategy against DCM and MI.

In summary, asprosin is a novel player in diabetes, obesity, PCOS, CVD; it is a molecule with broad research prospects. Asprosin may act as a biochemical marker for the diagnosis of diabetes and UAP as well as a therapeutic target for treating diabetes, obesity, DCM and ischemic cardiomyopathy based on the emerging evidence (Figure 2). Given that asprosin was only discovered in 2016, there are still many questions worthy of being addressed. These include the relationship between asprosin and obesity as well as PCOS, and the exact receptors and mechanisms underlying variable effects of asprosin on tissues and organs. In the future, asprosin as a therapeutic target may be utilized in clinical practice, but there is still a long way to go.

## Author Contributions

MY designed the study, MY, WL, and YZ drafted the manuscript. BY designed the figure. JW and MY revised the manuscript.

### Conflict of Interest

The authors declare that the research was conducted in the absence of any commercial or financial relationships that could be construed as a potential conflict of interest.
